# Temporal dynamics of the very premature infant gut dominant microbiota

**DOI:** 10.1186/s12866-014-0325-0

**Published:** 2014-12-31

**Authors:** Fabien Aujoulat, Laurent Roudière, Jean-Charles Picaud, Aurélien Jacquot, Anne Filleron, Dorine Neveu, Thierry-Pascal Baum, Hélène Marchandin, Estelle Jumas-Bilak

**Affiliations:** Université Montpellier 1, UMR 5119 ECOSYM, Equipe Pathogènes et Environnements Unité de Bactériologie, U.F.R. des Sciences pharmaceutiques et biologiques, 15, Avenue Charles Flahault, BP 14491, Montpellier, Cedex 5 34093 France; Centre Hospitalier de Fréjus, Laboratoire de bactériologie, 240 avenue de Saint-Lambert BP 110 83608, Fréjus, France; Hospices Civil de Lyon, Service de Néonatalogie, 103, Grande-Rue de la Croix-Rousse, Lyon, Cedex 04 69317 France; Centre Hospitalier Universitaire de Montpellier, Hôpital Arnaud de Villeneuve, Service de néo-natalogie, 371 Avenue du Doyen Gaston Giraud, Montpellier, Cedex 5 34295 France; Centre Hospitalier Régional Universitaire de Montpellier, Hôpital Lapeyronie, Département Urgences pédiatriques, 371, Avenue du Doyen Gaston Giraud, Montpellier, Cedex 5 34295 France; Centre Hospitalier Universitaire de Montpellier, Département d’Information Médicale, 371 Avenue du Doyen Gaston Giraud, Montpellier, Cedex 5 34295 France; Centre Hospitalier Universitaire de Montpellier, Hôpital Arnaud de Villeneuve, Laboratoire de Bactériologie, 371 Avenue du Doyen Gaston Giraud, Montpellier, Cedex 5 34295 France; Centre Hospitalier Universitaire de Montpellier, Laboratoire d’Hygiène hospitalière, 778, Rue de la Croix Verte, Montpellier, 34000 France

**Keywords:** Extremely low birth weight Infant, Stool, Microbiota, Follow-up, PCR-TTGE, Diversity, Dynamics, *Staphylococcus*, *Enterococcus*, *Clostridium*, *Enterobacteriaceae*, *Veillonellaceae*

## Abstract

**Background:**

The very-preterm infant gut microbiota is increasingly explored due to its probable role in the development of life threatening diseases. Results of high-throughput studies validate and renew the interest in approaches with lower resolution such as PCR-Temporal Temperature Gel Electrophoresis (TTGE) for the follow-up of dominant microbiota dynamics. We report here an extensive longitudinal study of gut colonization in very preterm infants. We explored by 16S rDNA-based PCR-TTGE a total of 354 stool specimens sampled during routine monitoring from the 1^st^ to the 8^th^ week of life in 30 very pre-term infants born before 30 weeks of gestational age.

**Results:**

Combining comparison with a diversity ladder and sequencing allowed affiliation of 50 Species-Level Operational Taxonomic Units (SLOTUs) as well as semi-quantitative estimation of Operational Taxonomic Units (OTUs). Coagulase-negative staphylococci, mainly the *Staphylococcus epidermidis*, was found in all the infants during the study period and was the most represented (75.7% of the SLOTUs) from the first days of life. Enterococci, present in 60% of the infants were early, highly represented and persistent colonizers of the premature gut. Later *Enterobacteriaceae* and the genus *Clostridium* appeared and were found in 10 (33%) and 21 infants (70%), respectively. We showed a high representation of *Veillonella* in more than a quarter of the infants and being able to persistently colonize premature gut. The genera *Anaerococcus*, *Aquabacterium*, *Bacillus*, *Bifidobacterium*, *Corynebacterium*, *Micrococcus*, *Oceanobacillus*, *Propionibacterium*, *Pseudomonas*, *Rothia*, *Sarcina*, *Sneathia* and *Streptococcus* were observed as transient or persistent colonizers, each genus being found in a minority of infants.

**Conclusions:**

Despite low resolution, PCR-TTGE remains complementary to high-throughput sequencing-based approaches because it allows the follow-up of dominant bacteria in gut microbiota in a large longitudinal cohorts of preterm neonates. We described the development of pre-term gut microbiota that should be now replaced regarding the functional role of major OTUs.

**Electronic supplementary material:**

The online version of this article (doi:10.1186/s12866-014-0325-0) contains supplementary material, which is available to authorized users.

## Background

Soon after birth, bacteria colonize the previously germ-free infant gastrointestinal tract. The colonizing bacteria participate to the barrier effect of the gut mucosa, facilitate carbohydrate assimilation, and modulate the immune system. Therefore, the establishment and dynamics of gut microbiota have a great influence on the healthy development of the infant. Microbiota composition and its dynamics have been described for healthy neonates [1,2] and to a lesser extent for pre-term neonates [3-5]. Knowledge regarding the gut microbiota dynamics in very premature infants (<30 weeks of gestation) remains limited whereas digestive complications are frequent and life-threatening in this population, particularly in the case of necrotizing enterocolitis (NEC) [6]. In addition, disequilibrium in the gut bacterial community might increase the risk of late-onset sepsis (LOS) after disruption of the immature mucosal barrier, resulting in translocation of luminal contents [7].

The impact of some bacterial species on the infant health and growth has been underlined [8]. For instance, De la Cochetière *et al.* showed by a molecular-based method that *Clostridium perfringens* appeared in the first 2 weeks of life in 3 infants who later developed a NEC [9]. More recently, unclassified bacteria of the family *Enterobacteriaceae* appeared to be associated with NEC [10]. On the opposite, bifidobacteria are considered to enhance installation and equilibrium of the microbiota, especially by maintaining the intestinal microbial balance and inhibiting the growth of pathogens [11,12]. However, the role of a single bacterium in health or diseases is doubtful [13,14] and one hypothesis is that inappropriate dynamics of gut colonization involving distortion and disequilibrium of the microbiota can cause NEC [8,10] and LOS [7].

Knowledge about the microbiota development arising from the chronological follow-up of extremely premature infants stool bacteria was initially based on culture studies. Among numerous studies, Sakata *et al*. (1985) [3] reported the longer study period (day 1 to day 49) but included only 7 infants. Other studies usually included more infants (26 to 99) with variable median gestational age (GA) (26 to 33 weeks) and follow-up period (one week to one month) [15-25]. Molecular methods have complemented or replaced cultural approaches for the study of complex ecosystems such as human resident microbiota [26]. The 16S ribosomal RNA (16S rRNA) gene-based PCR in association with sequence-specific separation tools such as T-RFLP (Terminal Restriction Fragment Length Polymorphism) [27,28], DGGE (Denaturing Gradient Gel Electrophoresis) [10,29-31] and TTGE (Temporal Temperature Gradient Gel Electrophoresis) [4,9,24,32,33] were used for studying bacterial gut communities in the premature population. More recently, studies based on pyrosequencing technology used deeper molecular analysis of the gut-associated microbiota of preterm infants. These studies revealed previously unappreciated biodiversity [5,31,34,35], including that of viruses and fungi [35]. However, this descriptive power is often underexploited, most high-throughput sequencing-based data being interpreted at the phylum level or with a focus on dominant Operational Taxonomic Units (OTUs) [10,31]. Exception is a recent metagenomic study that track strain level variations in 11 fecal samples chronologically collected in one premature infant [36 ref].

Considering dominant OTUs and high-level taxonomic ranks, fingerprint-based methods and high-throughput sequencing gave roughly congruent results [35]. Therefore, results of high-throughput studies validate approaches with lower resolution such as PCR-TTGE for dominant OTUs detection. PCR-TTGE allows a snapshot of the dominant microbiota and a monitoring of microbiota dynamics by identification of the dominant OTUs and analysis of multiple serial specimens in large population of preterm infants. We previously proposed an optimized approach for studying premature infant gut microbiota by PCR-TTGE and showed its reliability for bacterial identification and semi-quantitative estimation by comparison with culture [24]. This approach demonstrated a positive relationship between the diversity of the intestinal microbiota, digestive tolerance and weight gain in very preterm infants [33].

The aim of this longitudinal study was to describe the temporal dynamics of bacterial diversity in very preterm infant stools by a low-resolution focusing on dominant gut microbiota. Thirty infants born before 30 weeks of gestation were included, and a mean of 12 stool samples per infant were obtained chronologically from the 1^st^ to the 8^th^ week of life. A total of 354 stool samples were collected.

## Results and discussion

### Diversity of species-level operational taxonomic units

**Figure 1:**
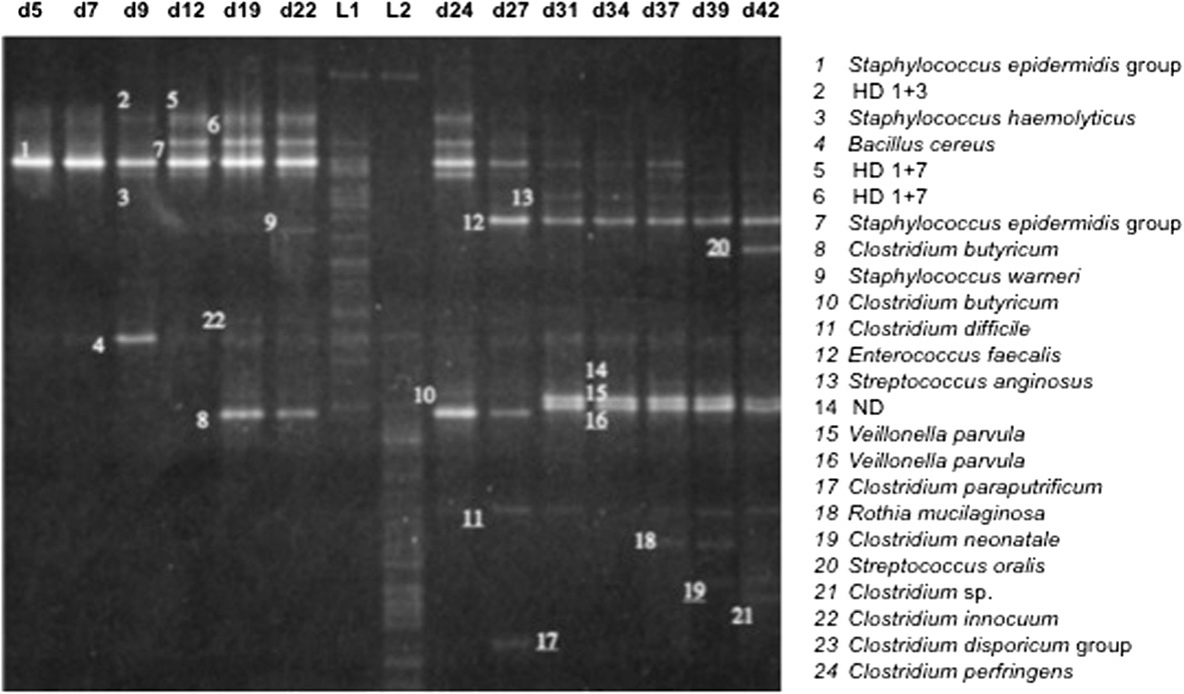
**Representative TTGE gel obtained for the chronological follow-up of patient D from day 5 (d5) to day 45 (d45).** Bands were identified with a number on the gel that corresponded to SLOTU identification. HD for heteroduplex band followed by the numbering of the bands forming the HD band. ND for not determined. L1 and L2 corresponded to the diversity ladders.

We analyzed 354 samples obtained from 30 patients. Figure 1 shows a representative TTGE gel corresponding to the chronological follow-up of the gut microbiota of the infant D. No DNA amplification was obtained for 14 samples from 11 infants (period of sampling: J3 to J23). Among the 1772 TTGE bands detected, 440 corresponded to artifactual bands, mainly heteroduplexes. This result underlined the high frequency occurrence of PCR artifacts when bacterial community was analyzed. If they remained unrecognized, they could lead to overestimated diversity. A total of 93 bands (7%) could not be analyzed because they did not correspond to bands present in the diversity ladder and because they were too faint to be cut and sequenced. Finally, 1239 bands were affiliated to a Species-Level Operational Taxonomic Unit (SLOTU). Our approach combining sequencing and comparison to a diversity ladder allowed artifact exclusion and SLOTU affiliation for about 93% of the bands with a sequencing effort of only 189 bands. We identified 50 different SLOTUs that reflected the overall microbiota diversity in 30 patients. The number of SLOTUs per patient varied from 0 to 13. The diversity was probably underestimated because among the 93 bands not affiliated to a SLOTU, 37 different distances of migration were distinguished. In a previous study associating TTGE and cloning/sequencing approaches, 288 clones were sequenced and 25 different SLOTUs were described [4]. A comprehensive culture-based study of the microbiota of preterm neonates of less than 1000 g surveyed during 30 days showed a total diversity of 20 bacterial species [19]. Comparatively with these previous approaches, we describe a wider bacterial diversity in stool samples of preterm neonates.

**Figure 2:**
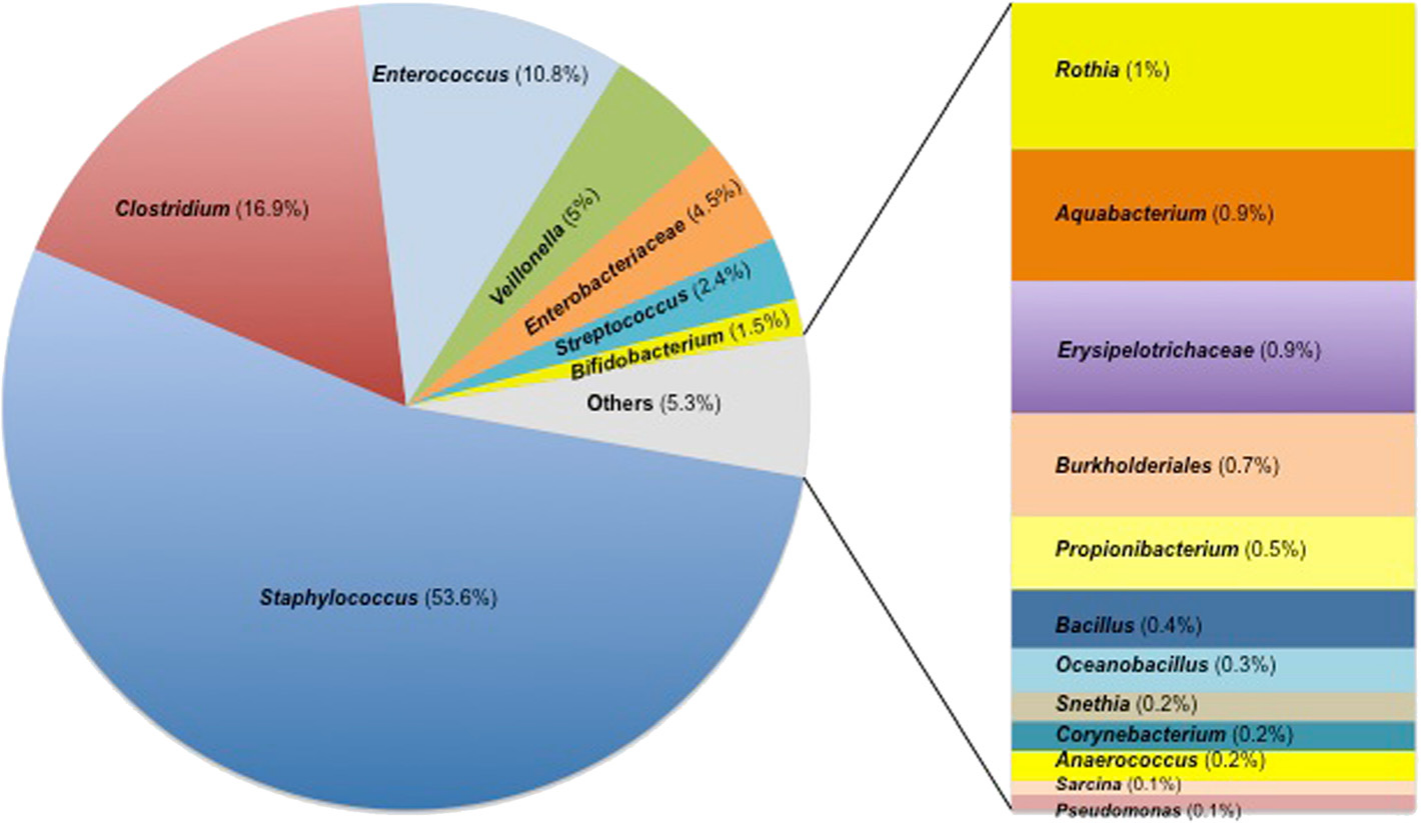
**Repartition in 19 GLOTUs determinated from SLOTU-assigned bands obtained by PCR-TTGE from 340 stool samples.**

The 50 SLOTUs were distributed in 19 genera or families named Genus-Level Operational Taxonomic Unit (GLOTU), which were defined on the basis of bacterial taxonomy (Table 1; Figure 2). The genera *Escherichia*, *Enterobacter* and *Klebsiella* were grouped in *Enterobacteriaceae* owing to the 16S rRNA gene relatedness among the genera of this family. The species *Clostridium innocuum* was affiliated to *Erysipelotrichaceae* because it does not belong to the genus *Clostridium* despite confusing nomenclature. The TTGE bands were distributed in each GLOTU as presented in Figure 2. Seven GLOTUs represented from 1.5% to 53.6% of total TTGE bands, *Staphylococcus*, *Clostridium Enterococcus*, *Veillonella*, *Enterobacteriaceae, Streptococcus* and *Bifidobacterium*. Each other GLOTU represented 1% or less of the total TTGE bands (Figure 2).

**Table 1:** **Frequency and semi-quantitative estimation of OTUs within samples and infants**

**Species-level OTU** ^**a**^ **(n = 50)**	**Genus and/or family-level OTUs (n = 19)**	**Samples with OTU n (%)** ^**c**^	**Infants with OTU n (%)** ^**d**^	**Period of detection (day)**	**Semi-quantitative score (band intensity)** ^**e**^		
					**1 (+) n (%) of bands**	**2 (++) n (%) of bands**	**3 (+++) n (%) of bands**
*Staphylococcus epidermidis* group*	*Staphylococcus*	**268 (75.7%)**	**30 (100%)**	d3-d53	45 (16.8%)	67 (25%)	**156 (58.2%)**
Staphylococcus haemolyticus*	*Staphylococcus*	185 (52.3%)	26 (86.7%)	d4-d51	34 (18.4%)	69 (37.3%)	**82 (44.3%)**
Staphylococcus warneri*	*Staphylococcus*	45 (12.7%)	16 (53.3%)	d3-d33	10 (22.2%)	16 (35.6%)	**19 (42.2%)**
Staphylococcus hominis*	*Staphylococcus*	13 (13.7%)	6 (20%)	d7-d45	4 (30.8%)	4 (30.8%)	**5 (38.4%)**
*Staphylococcus* sp.	*Staphylococcus*	6 (1.7%)	3 (10%)	d3-d25	2 (33.3%)	**3 (50%)**	1 (16.7%)
*Staphylococcus cohnii*	*Staphylococcus*	4 (1.1%)	1 (3.3%)	d27-d33	**4 (100%)**	0	0
ND^b^	Not determined	**93 (26.3%)**	**24 (80%)**	d3-d56	**60 (64.5%)**	16 (17.2%)	17 (18.3%)
*Enterococcus faecalis*	*Enterococcus*	**98 (27.7%)**	**18 (60%)**	d3-d56	13 (13.3%)	19 (19.4%)	**66 (67.3%)**
*Enterococcus durans* group	*Enterococcus*	7 (2%)	2 (6.7%)	d31-d53	1 (14.3%)	2 (28.6%)	**4 (57.1%)**
Clostridium butyricum*	*Clostridium/Clostridiaceae*	**40 (11.3%)**	**12 (40%)**	d18-d53	8 (20%)	11 (27.5%)	**21 (52.5%)**
Clostridium difficile*	*Clostridium/Clostridiaceae*	34 (9.6%)	11 (36.7%)	d24-d56	14 (41.2%)	**17 (50%)**	3 (8.8%)
*Clostridium neonatale*	*Clostridium/Clostridiaceae*	29 (8.2%)	10 (33.3%)	d16-d53	**12 (41.4%)**	**12 (41.4%)**	5 (17.2%)
*Clostridium perfringens*	*Clostridium/Clostridiaceae*	23 (6.5%)	9 (30%)	d17-d50	8 (34.8%)	4 (17.4%)	**11 (47.8%)**
*Clostridium* sp.	*Clostridium/Clostridiaceae*	9 (2.5%)	4 (13.3%)	d27-d42	**6 (66.7%)**	1 (11.1%)	2 (22.2%)
*Clostridium paraputrificum*	*Clostridium/Clostridiaceae*	5 (1.4%)	3 (10%)	d15-d18	**2 (40%)**	**2 (40%)**	1 (20%)
*Clostridium tertium*	*Clostridium/Clostridiaceae*	9 (2.5%)	3 (10%)	d22-d49	3 (33.3%)	3 (33.3%)	3 (33.3%)
*Clostridium disporicum* group	*Clostridium/Clostridiaceae*	5 (1.4%)	2 (6.7%)	d15-d24	**2 (40%)**	**2 (40%)**	1 (20%)
*Clostridium corinoforum*	*Clostridium/Clostridiaceae*	1 (0.3%)	1 (3.3%)	d34	**1 (100%)**	0	0
Clostridium glycolicum*	*Clostridium/Clostridiaceae*	1 (0.3%)	1 (3.3%)	d50	**1 (100%)**	0	0
*Clostridium favososporum*	*Clostridium/Clostridiaceae*	5 (1.4%)	1 (3.3%)	d31-d50	**3 (60%)**	1 (20%)	1 (20%)
*Clostridium symbosium* group	*Clostridium/Clostridiaceae*	2 (0.6%)	1 (3.3%)	d21-d27	0	**1 (50%)**	**1 (50%)**
*Sarcina ventriculi*	*Sarcina/Clostridiaceae*	1 (0.3%)	1 (3.3%)	d17	0	0	**1 (100%)**
Escherichia coli*	*Enterobacteriaceae*	**22 (6.2%)**	**8 (26.7%)**	d6-d56	**11 (50%)**	0	**11 (50%)**
*Klebsiella oxytoca*	*Enterobacteriaceae*	13 (3.7%)	4 (13.3%)	d12-d56	4 (30.8%)	1 (7.7%)	**8 (61.5%)**
*Klebsiella pneumoniae*	*Enterobacteriaceae*	3 (0.8%)	2 (6.7%)	d39-d49	0	0	**3 (100%)**
*Enterobacter cloacae*	*Enterobacteriaceae*	5 (1.4%)	1 (3.3%)	d38-d56	1 (20%)	0	**4 (80%)**
*Veillonella dispar*	*Veillonella*	**31 (8.8%)**	**6 (20%)**	d4-d56	**20 (64.5%)**	3 (9.7%)	8 (25.8%)
*Veillonella parvula*	*Veillonella*	9 (2.5%)	3 (10%)	d28-d45	1 (11.1%)	**5 (55.6%)**	3 (33.3%)
*Veillonella* sp.	*Veillonella*	7 (2%)	2 (6.7%)	d19-d42	2 (28.6%)	2 (28.6%)	**3 (42.8%)**
*Rothia mucilaginosa*	*Rothia*	10 (2.8%)	4 (13.3%)	d37-d52	3 (30%)	3 (30%)	**4 (40%)**
Aquabacterium commune*	*Aquabacterium/Burkholderiales*	**7 (2%)**	**4 (13.3%)**	d3-d41	**5 (71.4%)**	2 (28.6%)	0
Aquabacterium citratiphilum*	*Aquabacterium/Burkholderiales*	2 (0.6%)	1 (3.3%)	28-d30	**1 (50%)**	**1 (50%)**	0
*Burkholderiales* ND	*Burkholderiales*	7 (2%)	1 (3.3%)	d16-d33	**5 (71.4%)**	1 (14.3%)	1 (14.3%)
*Streptococcus oralis* group	*Streptococcus*	4 (1.1%)	**3 (10%)**	d24-d50	**2 (50%)**	1 (25%)	1 (25%)
*Streptococcus salivarius*	*Streptococcus*	**10 (2.8%)**	2 (6.7%)	d31-d56	**5 (50%)**	**5 (50%)**	0
*Streptococcus anginosus*	*Streptococcus*	1 (0.3%)	1 (3.3%)	d3	0	0	**1 (100%)**
*Streptococcus oralis*/*parasanguinis*	*Streptococcus*	1 (0.3%)	1 (3.3%)	d49	**1 (100%)**	0	0
*Streptococcus parasanguinis* group	*Streptococcus*	1 (0.3%)	1 (3.3%)	d42	0	**1 (100%)**	0
*Streptococcus sanguinis* group	*Streptococcus*	1 (0.3%)	1 (3.3%)	d28	**1 (100%)**	0	0
*Streptococcus thermophilus*	*Streptococcus*	5 (1.4%)	1 (3.3%)	d16-d28	**2 (40%)**	1 (20%)	**2 (40%)**
*Bacillus cereus* group	*Bacillus/Bacillaceae*	**4 (1.1%)**	**4 (13.3%)**	d9-d56	**3 (75%)**	0	1 (25%)
*Oceanobacillus* sp.	*Oceanobacillus/Bacillaceae*	3 (0.8%)	1 (3.3%)	d18-d24	**3 (100%)**	0	0
Bifidobacterium breve*	*Bifidobacterium*	**9 (2.5%)**	**2 (6.7%)**	d3-d49	3 (33.3%)	**6 (66.7%)**	0
*Bifidobacterium longum*	*Bifidobacterium*	5 (1.4%)	2 (6.7%)	d22-d50	**3 (60%)**	2 (40%)	0
*Clostridium innocuum*	*Erysipelotrichaceae*	9 (2.5%)	1 (3.3%)	d10-d36	3 (33.3%)	**6 (66.7%)**	0
*Propionibacterium* sp.	*Propionibacterium*	**3 (0.8%)**	1 (3.3%)	d18-d24	1 (33.3%)	0	**2 (66.7%)**
*Propionibacterium acnes*	*Propionibacterium*	2 (0.6%)	1 (3.3%)	d43-d45	0	**2 (100%)**	0
*Sneathia sanguinegens*	*Sneathia*	2 (0.6%)	1 (3.3%)	d3-d4	**1 (50%)**	0	**1 (50%)**
*Pseudomonas* sp.	*Pseudomonas*	1 (0.3%)	1 (3.3%)	d37	0	**1 (100%)**	0
*Anaerococcus octavius*	*Anaerococcus*	2 (0.6%)	1 (3.3%)	d32-d37	0	**2 (100%)**	0
*Corynebacterium tuberculostearicum*	*Corynebacterium*	2 (0.6%)	1 (3.3%)	d14-d15	0	**2 (100%)**	0

^a^OTUs are indicated by main groups of decreasing occurence among the 30 preterm infants.

^b^ND, OTU not determined (not included in the total SLOTU number of 50): the group included bands with 37 distinct migration distances.

^c^Number and % of samples with OTU among the 354 samples, bold type indicates the species-level OTU mostly represented in samples within the corresponding genus and/or family-level OTU.

^d^Number and % of preterm infants with OTU among the 30 neonates, bold type indicates the species-level OTU mostly represented in neonates within the corresponding genus and/or family-level OTU.

^e^Bold type indicates the most frequent semi-quantitative score observed for the OTU within PCR-TTGE patterns.

*OTU identified in patient ZA with NEC.

As expected, pyrosequencing approaches detected more SLOTUs but most of them belonged to minority populations, each representing less than 1% of the total diversity [35]. Again, higher genus-level diversity was reported with high-throughput sequencing methods revealing up to 365 genera in the fecal microbiota of premature infants [7,10]. However, LaTuga *et al*. [35] showed that 1 to 2 genera out of the 61 found in their study comprised over 90% of the sequences in most individual samples [35].

**Figure 3:**
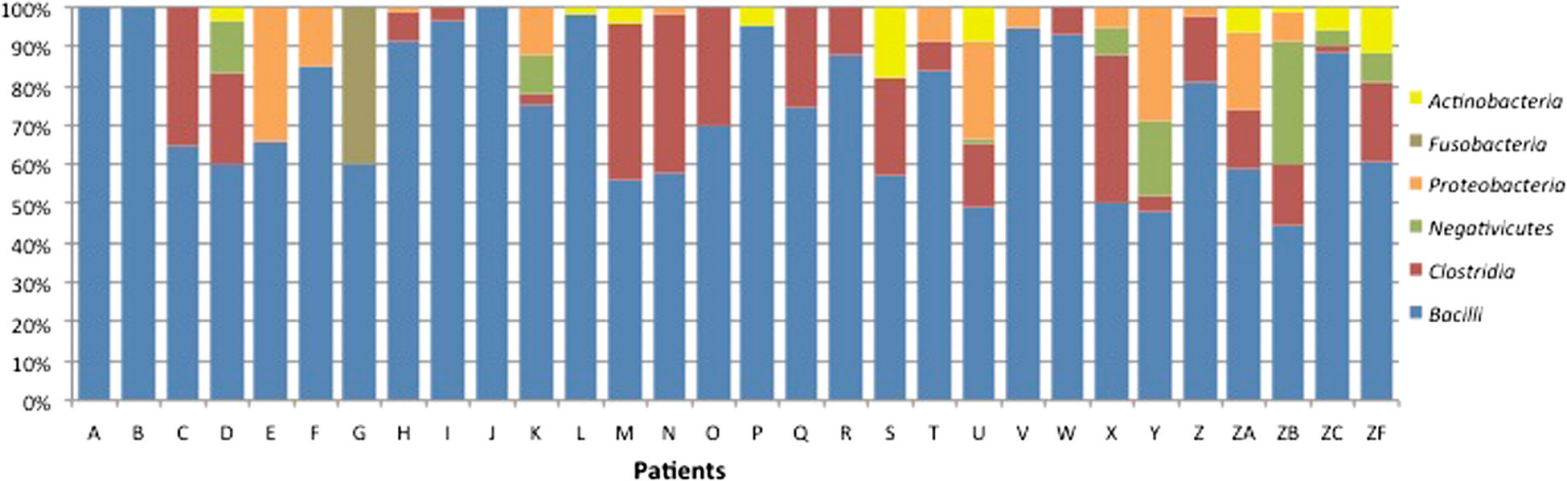
**Relative abundance of high-level taxa per patient.**

To determine the relative repartition of high-level taxa per patient, the GLOTUs were grouped in their corresponding phylum or class for the phylum *Firmicutes* to form PLOTUs (Phylum-Level Operational Taxonomic Units). The bacteria described belonged to the 6 following PLOTUS: phyla *Actinobacteria*, *Proteobacteria*, *Fusobacteria* and classes *Bacilli, Clostridia* and *Negativicutes* in the phylum *Firmicutes*. Although most patients carried only 2 to 4 PLOTUs, we observed important variations among patients (Figure 3). The *Firmicutes* represented the large majority of SLOTUs in all patients and 9 patients harboured only *Firmicutes. Bacilli* was the sole and generally predominating PLOTU found in all neonates and was the only PLOTU recovered in 3 patients. Beside *Bacilli*, *Clostridia* was the most represented PLOTU found in 21 neonates. Finally, Gram-positive bacteria largely dominated the bacterial community since they represented 88% of the identified SLOTUs.

High-throughput sequencing methods revealed higher diversity than we observed herein but the 3 dominant phyla (*Firmicutes*, *Actinobacteria*, *Proteobacteria*) were concordantly found [5,7,10,35]. However, discrepancies between studies were observed for the phylum *Bacteroidetes*, most often found as a rarely represented phylum while representing about 8% of the sequences in the study by Mai *et al.* [7]. Surprisingly, members of the phylum *Fusobacteria* found in our study were not reported by these sequencing-based studies [5,7,10,35]. The phylum diversity was not markedly higher in the gut microbiota of adults and infants. Ten phyla were detected in the stool specimens of the 242 adults included in the Human Microbiome Project but this number generally decreases to 3 to 4 phyla if each subject is considered [37].

In adults and weaning children, *Bacteroidetes* is the major bacterial phylum constituent of the gut microbiota followed by *Firmicutes* such as *Eubacterium*, *Ruminococcus* and *Clostridium*. In the extreme premature neonates studied herein, *Firmicutes* appeared the major component of the gut microbiota but *Bacteroidetes* were unrepresented (Figure 2). Members of *Firmicutes* differed from those described in adults since the class *Bacilli* with the genera *Staphylococcus*, *Streptococcus* and *Enterococccus* was the most represented. Members of the class *Negativicutes* were also well represented.

Finally, although PCR-TTGE lacks sensitivity in the detection of minority population, major GLOTUs and PLOTUs were detected in accordance with molecular methods with higher throughput.

### Dynamics of extremely premature infant gut microbiota

**Figure 4:**
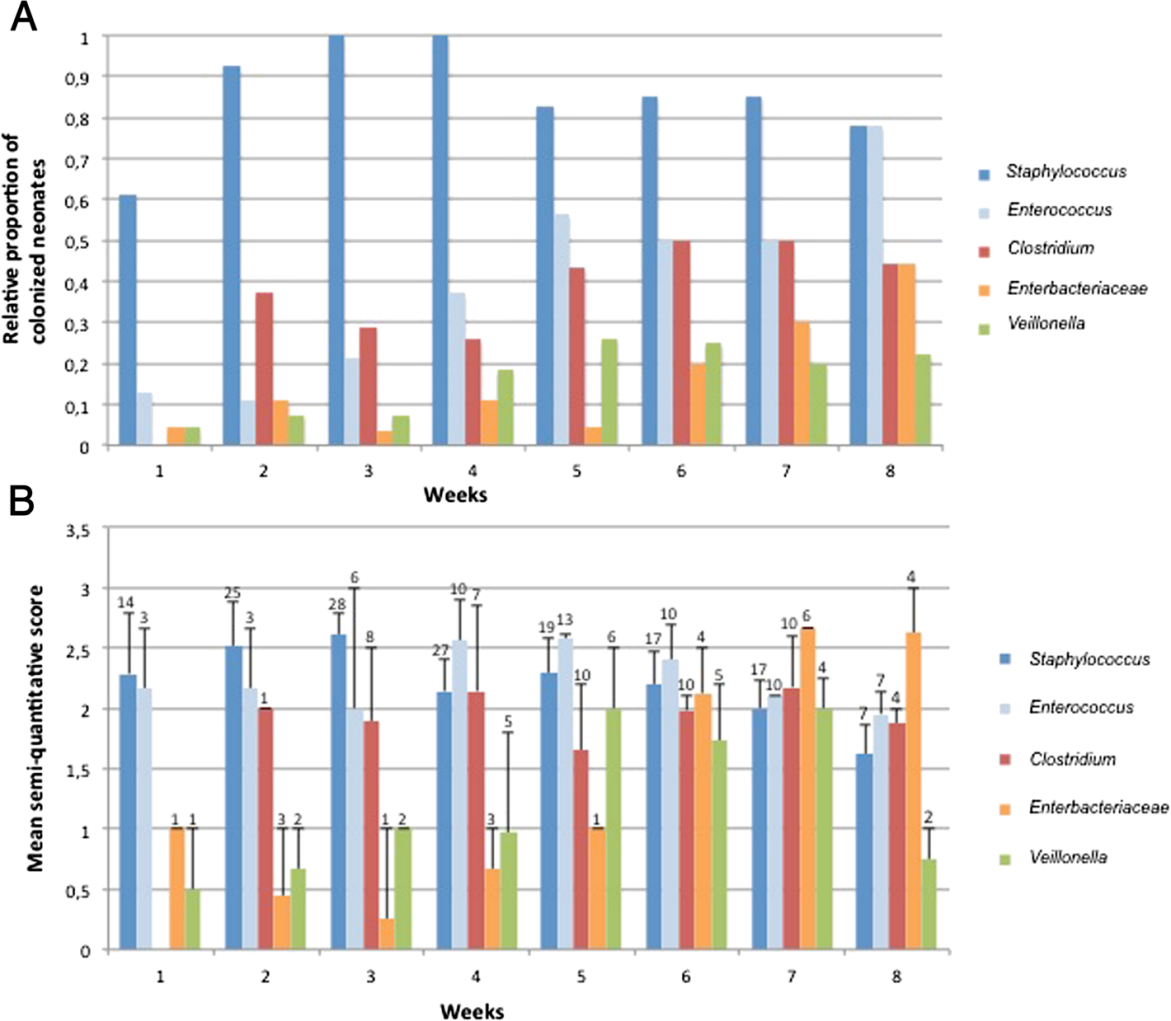
**Dynamics of the 5 main GLOTUs in the gut flora over the time of survey (8 weeks). A)** proportion of neonates colonized by each GLOTUs. **B)** semi-quantitative development of each GLOTUs in the sub-population of neonates colonized by this GLOTU indicated as mean semi-quantitative score per week. Bars indicate mean maximal semi-quantitative score observed among the population colonized with a GLOTU and numbers above bars the number of colonized preterm neonates by the considered GLOTU.

We studied the repartition of the 5 major GLOTUs per infant and per age post-delivery. The Figure 4A shows the relative proportion of infants displaying each major GLOTU and the weekly development of the GLOTUs’ occurence. The only GLOTU shared by all the infants and found in more than 75% of the analysed samples was *Staphylococcus* spp. The percentage of infants colonized with enterococci, clostridia and enterobacteria increased over the survey period, markedly towards the 5^th^ week of life when the percentage of neonates carrying staphylococci began to decrease. The increase in members of the family *Enterobacteriaceae* was slightly delayed compared to the Gram-postive bacteria, enterococci and clostridia. In a previous study, we showed a global congruence between a semi-quantitative score calculated from intensity of TTGE bands and the number of colony-forming units (CFU), thereby validating the semi-quantitative approach based on band intensity [24]. We explored here the semi-quantitative dynamics of the major GLOTUs detected in the gut flora of the 30 neonates (Figure 4B). We showed that *Staphylococcus*, *Enterococcus* and *Enterobacteriaceae* successively dominated the very preterm neonate gut microbiota. Indeed, the semi-quantitative approach showed that *Staphylococcus* was the predominant population for the first 3 weeks of life followed by enterococci from the 4^th^ to the 6^th^ week of life and then by enterobacteria (Figure 4B). Semi-quantification of *Clostridium* showed they did not vary significantly over time (Figure 4B). Detailed microbiota composition over time is given for the 30 patients in the Additional file 1: Table S1 together with semi-quantitative scores deduced from band intensity for each OTU occurrence (Additional file 1: Table S1). Less than 25% of the patients carried each minor GLOTU whatever the date after delivery and their qualitative or semi-quantitative (Additional file 1: Table S1) representation did not change significantly with the date of sampling.

### Staphylococci dominate the extremely premature infants gut microbiota

The genus *Staphylococcus* represented 53.6% of bands affiliated to a SLOTU (Figure 2). All infants carried *Staphylococcus* spp. at least once over the follow-up period. Up to four staphylococcal OTUs may be found in an infant (n = 4, 13.3% of the infants) but successive or concomitant colonization by 3 OTUs was observed in most infants (n = 17, 57%). For most neonates, staphylococci appeared during the first week of life and persisted up to the 8^th^ week post-delivery with a slight decrease in colonized infants after the 4^th^ week (Figure 4A). The semi-quantitative approach showed that staphylococci quantitatively dominate the gut microbiota up to the third week of life (Figure 4B). Most of the staphylococci were coagulase-negative staphylococci (CoNS) belonging to the *S. epidermidis* genomic group defined by DNA-DNA hybridization and 16S rRNA gene phylogeny that included *S. epidermidis*, *S. caprae, S. capitis, S. aureus* and *Staphylococcus warneri* [38,39]*.* In this group of genetically very related species, *S. warneri* displayed particular TTGE behaviour and therefore could be identified separately. Different behaviours were observed for the CoNS species found in this study. Contrarily to other species, *S. warneri* was only found in the first month of life (Table 1). Colonization by a CoNS species may be transient as observed for *S. warneri* but also for *S. hominis* and *Staphylococcus cohnii* while *S. epidermidis* and *S. haemolyticus* usually appeared as persistent colonizers over the study period. After first detection, these two species were indeed found in more than 50% of the subsequent samples in 29 out of the 30 and in 20 out of the 26 infants colonized by each species, respectively.

CoNS predominance could be considered as a specific trait of the extremely premature infant gut microbiota fed by human pasteurised milk since staphylococci appeared as a minor or an unrepresented population in the gut community of adults [40,41] or infants born at term, whatever their feeding pattern [2,41]. CoNS have received a marginal attention for their role in the early colonization of the premature and extremely premature infant gut microbiota. Few studies reported the detection of CoNS in neonate stools by culture, with controversial results concerning the load and the prevalence of these bacteria [3,42,43]. Molecular-based studies also detected staphylococci that seemed to dominate the extremely premature infant gut microbiota in the first weeks of life [4,9]. In these studies, predominance of CoNS was observed in only few cases due a low number of extremely preterm neonates included [9] or to the low sampling effort in each patient [4].

CoNS also retained attention since they are the major cause of LOS in neonatal intensive care unit (NICU) [44]. CoNS were supposedly acquired postnatally [44] and sometimes during nosocomial outbreaks [45]. The gut has recently been described as a reservoir of CoNS implicated in late-onset infections of extremely premature infants [46] and distortion in gut microbiota seemed to be related to LOS [7]. The breast-feeding origin of the neonate gut colonization by CoNS has been suspected because of the high prevalence of *S. epidermidis* in the milk of lactating women [47].

### *Clostridium* spp

16.9% of the TTGE bands obtained in this study corresponded to sequences affiliated to *Clostridium* spp. The genus *Clostridium* appeared as the most diverse since it was represented by 12 different SLOTUs (Table). *Clostridium difficile* (34 samples from 11 patients), *Clostridium neonatale* (29 samples from 10 patients), *Clostridium butyricum* (40 samples from 12 patients) and *Clostridium perfringens* (23 samples from 9 patients) were the most represented SLOTUs. Each other minor *Clostridium* SLOTU was found in 1 to 4 patients. Infants may be colonized by up to 5 clostridial OTUs (n = 4) but more frequently harboured 2 (n = 5) to 3 (n = 6) OTUs during the study period. *Clostridium* represented qualitatively a major group among the premature neonate gut microbial community (Figure 4A), colonizing 70% (n = 21) of the infants over the study period. The percent of *Clostridium* colonization of premature neonates is congruent with the result obtained by stool culture (79%) [48]. The colonization began on the second week of life, then the number of colonized infants increased regularly from the 4^th^ to the 6^th^ week of life reaching 50% of neonates colonized by *Clostridium* spp. The semi-quantitative approach showed that *Clostridium* spp. represented a relatively important bacterial population as soon as they appeared and that bacterial load appeared stable over the survey period (Figure 4B). Considering OTUs shared by 2 infants or more, transient or persistent colonization was observed for the major clostridial OTUs (*C. butyricum*, *C. difficile*, *C. neonatale* and *C. perfringens*) and *Clostridium tertium* depending on the infants while *Clostridium paraputrificum* and *Clostridium disporicum* group appeared as transient colonizers only.

*C. difficile* is a recognized pathogen since the toxinogenic strains cause severe enterocolitis and antibiotic-associated diarrhoea. The species represent up to 50% of the bacterial species isolated from infants, particularly from formula-fed infants [1]. In breast-fed infants *C. difficile* represented from 6% to 20% of the cultivated micro-organisms [1]. Healthy neonates may harbour *C. difficile* and its toxins without apparent pathologic consequences [42]. In our population, about 1/3 of very preterm neonates were colonized by *C. difficile*. “*Clostridium neonatale*” is a non-validated species described during a NICU outbreak where 20.8% of neonates carried the same “*C. neonatale*” strain [49]. We showed that out of an epidemic context, 1/3 of our patients were colonized by *C. neonatale*. The role of *C. neonatale* and its specificity for the neonate microbiota need to be explored. *Clostridium* spp., particularly *C. perfringens* [9], *C. difficile* [50] and *C. butyricum* [48,51] have been related to NEC. We showed that these OTUs frequently colonized the neonates included in this study. One patient developed a NEC, *C. butyricum* was detected twice, at the onset of the clinical signs and at day 31 after the onset of NEC (patient ZA).

### *Enterococcus* spp

SLOTUs affiliated to the genus *Enterococcus* were among the most represented (10.8%) and were found in 18 out of the 30 infants (60%). However, diversity of enterococcal OTUs was rather low with 2 SLOTUs found during the study: *Enterococcus faecalis* which was the most represented and *Enterococcus durans* group (Table). Among the population, only 2 patients (6.7% of the infants) were successively or simultaneously colonized by these 2 SLOTUs. At the first week of life, 13% of the patients were colonized. The level of colonization increased regularly over time, about half of the patients being colonized at the 5^th^ week of life and up to 77% at the 8^th^ week (Figure 4A). The semi-quantitative evaluation showed that enterococci were the second dominating genus of the very premature gut microbiota after staphylococci up to the 3^rd^ week of life. Then, enterococci became quantitatively the major population during the following 3 weeks of life.

Despite inter-individual variations [2], enterococci were found among the main groups encountered in the gut microbiota of preterm infants [4] and represented one of the main groups during initial colonization in healthy infants in other molecular-based studies [27]. As observed herein, culture-based studies previously showed early enterococcal colonization that increased over time with a dynamic roughly similar to enterobacteria colonization (Figure 4A) [52], even if the quantitative increase in enterobacteria was delayed relative to enterococci (Figure 4B). However, in our population, the level of colonization was slightly lower than previously described in culture-based studies. For example, enterococcal colonization was shown by Hufnagel *et al.* (2007) [52] in 23% of the patients admitted to a NICU. In another instance, at the end of the first week of life, enterococci were isolated from stools of a large majority (87%) of the preterm infants [17]. Persistant colonization was the most frequent finding for *Enterococcus* OTUs found in this study, observed in 83% of the infants harbouring *E. faecalis* and in the two infants colonized by *E. durans* group.

Both prematurity and low birth weight were statistically associated with enterococcal colonization on the first day of life [17]. Influence of the diet was also reported with significantly higher enterococcal counts for formula-fed infants compared to breast milk-fed infants [53]. Although enterococci were responsible for life-threatening infections, enterococcal colonization on the first day of life was not associated with differences in the outcome of infants with respect to death and life-threatening diseases [52].

### The genus *Veillonella*

The genus *Veillonella* was the only representative of the class *Negativicutes* detected in this study. Five percent of the TTGE bands were affiliated to the genus *Veillonella* (Figure 2). Eight out of the 30 patients (26.6%) were colonized with *Veillonella* at least once during the survey (5 infants by one OTU and 3 by 2 OTUs). About 5% of the neonates were colonized during the first week of life, this rate reaching about 20% from the 4^th^ week of life. Semi-quantitatively, high representation of *Veillonella* was observed between the 5^th^ and the 7^th^ week of life (Figure 4B). Large inter-individual variation was noted since *Veillonella* spp.-corresponding TTGE bands accounted for less than 5% to more than 30% of the generated bands depending on the patient (Figure 3). We showed here that members of the genus *Veillonella* could be present in gut microbiota of very premature infants, sometimes with high representation (patients D, Y and ZB in Figure 3) and persistent after the first colonization (patients D, K, X, Y and ZB) (Additional file 1: Table S1).

*Veillonella* spp. is part of several normal human microbiota including the fecal microbiota of healthy adults [54] and is predominant in the upper digestive tract [55]. Variations in the count of *Veillonella* spp. were previously noted depending on diet, pathology or antimicrobial treatment [56,57]. *Veillonella* may play a role in gut microbiota development at an early age [58] since they were shown to appear in neonate faeces on the first days of life [2,59], with significantly higher count in bottle-fed than in breast-fed children [1].

In oral flora, *Veillonella* spp. are early colonizers [60] establishing a nutrition chain with other bacteria [61] and are statistically associated with periodontal health [62]. *Veillonella* obtain energy from lactic acid produced by the streptococci as an end product of carbohydrate fermentation [63]. By analogy, one can expect that such a metabolic interaction between *Veillonella* spp. and lactic acid producers, notably staphylococci and streptococci, the main representatives of the major class *Bacilli* in this study, may occur in the digestive tract. The role and the impact of such a potential metabolic cooperation between early colonizers of the gut microbiota of premature infants are worth to be investigated.

### *Enterobacteriaceae*

The family *Enterobacteriaceae* was represented by 4.5% of the TTGE bands observed in this study (Figure 2) and was demonstrated in 10 infants (33.3%) during the study period. Most colonized patients (n = 6, 60%) harbored a unique enterobacterial OTU while 3 OTUs were recovered during the follow-up of patient Y. The representation of this family was lower than observed in culture-based studies [17] as well as in previous molecular-based studies, 36% of the clones detected in the study of Magne *et al.*, [4] being affiliated to *Enterobacteriaceae*. In our patient population, enterobacteria were found in about 5 to 10% of the neonates during the 5 first weeks of life, then the rate of colonization increased to reach 45% of the neonates at the 8^th^ week (Figure 4A). Semi-quantitatively, we observed a rapid increase in the population after the 30^th^ day of life, enterobacteria being the dominating bacteria of the microbiota from the 7^th^ week of life (Figure 4B). Four SLOTUs were retrieved, *Escherichia coli, Klebsiella pneumoniae, Klebsiella oxytoca* and *Enterobacter cloacae. E. coli* was the main species represented, accounting for more than half of the SLOTUs of the family *Enterobacteriaceae* and was found in 8 preterm neonates. *Klebsiella* spp. and *Enterobacter cloacae* were found in 6 and 1 patients, respectively, and always showed a complex TTGE profile composed of several bands probably corresponding to 16S rDNA intragenomic variability as previously described [29].

Most of the TTGE bands (86%) corresponding to *Enterobacteriaceae* were observed in samples obtained after the 25^th^ day of life and it was only after the 30^th^ day of life, when the population quantitatively increased, that we observed *Enterobacteriaceae* in several successive samples. This suggests a durable implantation of enterobacteria after the first month of life of very premature neonates contrary to the early implantation described in healthy infant [2,64] and in pre-term infant gut [3].

### Minority populations

Fourteen minor GLOTUs, each representing less than 2.4% of the total GLOTUs (Figure 2), were detected in this study. Generally, less than 10% of the patients were colonized with such minor groups whatever the week of sampling (Additional file 1: Table S1) and semi-quantitative analysis showed that these groups were present at low load in the microbiota (data not shown). They included 23 SLOTUs, some of them being found in only one sample (*Sarcina ventriculi*, *Bacillus* sp. *Oceanobacillus* sp. etc.). Others were able to colonize durably the neonate gut and were detected in several chronological samples in one or more patient. This was the case for *Bifidobacterium breve* (patient ZF), *Bifidobacterium longum* (patient U), *Rothia mucilaginosa* (patient S), *Streptococcus salivarius* (patients X and Y), and *Streptococcus thermophilus* (patient T), which were previously described in neonate gut microbiota.

The genus *Streptococcus* appeared as a major group in the study of Magne *et al.* [4] whereas in accordance with our results streptococci were seldom detected in other studies [16,17,29]. Moreover, streptococcal OTUs were most often transient colonizers of the neonatal gut except for *S. salivarius* in patients X and Y.

Bifidobacteria were detected in 4 patients (14 samples) and represented less than 1% of the TTGE bands analysed in this study. The rarity of bifidobacteria in our population of very preterm infants confirmed previous results [4]. Bifidobacteria were described as early and dominant colonizers of healthy full-term neonates specially breast-fed neonates [1,58,65], but their colonization is delayed in pre-term infants [3,16,17]. In a recent study, probiotics, in the form of *Bifidobacterium* and *Lactobacillus*, fed enterally to very low birth weight preterm infants for 6 weeks were shown to reduce the incidence of death and necrotizing enterocolitis [66]. The low prevalence of bifidobacteria in very preterm neonate gut could be an additional argument for their use as probiotics. However, the patient ZA who developed a NEC was one of the four patients colonized by bifidobacteria in this study. We did not detect lactobacilli in this study.

To our knowledge, *Burkholderiales* (including *Aquabacterium commune* and *Aquabacterium citraphilum*), *Sneathia sanguinegens* and *Rothia mucilaginosa* were described here for the first time as colonizers of the neonate gut. Of note, *A. commune* and *A. citraphilum* have been found in four samples from the patient ZA, who developed a NEC. Some patients displayed very atypical microbiota; for instance, the patient T showed a persistent gut colonization by *Streptococcus thermophilus* and an unidentified *Burkholderiales* beside staphylococci over a 15-day period. No clinical or epidemiological particularity was noted for this neonate. The patient G was colonized by staphylococci, *Snethia sanguinegens* (*Fusobacteria*), and an unidentified taxon and died at day 9 of life due to a non-digestive cause (Figure 3).

### Conclusions

Today, pyrosequencing and other high-throughput sequencing methods allow the in-depth description of microbiota. However, high throughput metagenomics is until today limited to a relative low number of samples [5,7,10,35], as examplified in recent matagenomic studies of the neonate gut microbiota. Despite low resolution, fingerprinting-based methods remain complementary of sequencing-based approaches because they allowed the follow-up of the dominant gut microbiota in larger cohorts of patients.

We describe here bacterial colonization dynamics in consecutive stool samples of 30 very premature infants. Gram-positive bacteria (staphylococci, enterococci and clostridia) represent the quantitatively dominant microbiota established in the neonate gut during the first month of life, staphylococci being the earliest colonizers found in a large majority of infants. Gram-negative bacteria (*Enterobacteriaceae* and *Veillonella*) established a delayed and inconstant colonization over the study period compared to Gram-positive bacteria.

Beside dominant microbiota, satellite taxa are numerous, generating a great interindividual variability in the composition and chronology of colonizing species, despite a monocentric recruitment, like in term neonates. According to the literature, this variability has to be related to multiple factors such as GA, mode of delivery, birth weight, per partum maternal antibiotherapy and early neonatal antibiotherapy. Thus, defining the normal gut-associated microbiota in preterm infants remains a challenge but is a prerequisite not only for future therapy aiming to intestinal microbiota modification but also for future research on intestinal diseases of the neonate.

## Methods

### Population and stool sample collection

We included 30 very preterm infants, born between 26 and 29 weeks of GA (median value = 27) with a median birth weight of 950 g (760 g - 1060 g) and hospitalized in the NICU of the Montpellier university hospital. Stool samples were prospectively collected once to twice a week in each patient between day 3 and day 56 of life or until the last day of stay in the neonatal unit. Four to 15 samples were obtained for each patient (mean value = 11.8). Other clinical characteristics recorded for the population were reported elsewhere [33]. Formal ethical approval was not required for this study but a written informed consent for participation in the study was obtained from the patient’s parents.

### DNA extraction from stool sample

Stool samples (50 mg, wet weight) were suspended in 1 mL of sterile DNA-free water and homogenized. The suspension was centrifuged at 10,000 g for 10 min at room temperature. The supernatants and the fat layers were removed. The pellets were suspended in 150 μl of Tris-EDTA buffer and DNA was extracted using the MasterPure Gram positive DNA purification kit (Epicentre) according to supplier’s instructions but with lysozym incubation prolonged to 16 hours.

### PCR-TTGE assays

The V2-V3 region of the 16S rRNA gene was amplified with the primers HDA1 (5’-ACTCCTACGGGAGGCAGCAGT-3’) and HDA2 (5’-GTATTACCGCGGCTGCTGGCA-3’) (Ogier, 2002) giving a PCR product of about 233 bp. A GC-clamp 5’-CGCCCGGGGCGC GCCCCGGGCGGGGCGGGGGCACGGGGGG-3’ was added at the 5’ side of the primer HDA1 for PCR-TTGE analysis. The PCR was performed with FastStart High fidelity PCR system (Roche). PCR and TTGE conditions were previously described [24].

### TTGE analysis and band identification

The gel migration distance of TTGE bands was measured manually. Bands were assigned to OTUs either by comparison to a homemade « TTGE diversity» ladder which includes fingerprints of pure strains isolated from infant stools or by sequencing. The ladder was constructed as previously described [24]. Band comparisons were performed after gel standardization using three internal migration standard loaded with DNAs amplified from samples. Heteroduplex artifacts corresponding to chimeric amplification with each DNA strand coming from different bacteria in the community. Heteroduplex bands generally appeared as thin and/or faint bands at the top of the gel or nearly up to an intense band. Band sequencing confirmed their heterogeneous nature.

For sequencing, bands obtained from the TTGE gels were excised, washed three times in sterile distilled water, and incubated overnight in 50 μL of elution buffer (Qiagen). The supernatant was used in a PCR experiment performed as described above with the primers HDA1 without GC-clamp and HDA2. Amplified DNA was then sequenced on an ABI 32 sequencer (Beckman Coulter Cogenics). The sequences were compared to the NCBI, RDPII and Greengenes databases to determine OTUs (Additional file 2). Each sequence was affiliated to a SLOTU that corresponded to a phylotype, i.e., a terminal node in 16S rRNA-based phylogenetic analysis. In most cases, one SLOTU corresponded to one species, but it could correspond to a group of few species not separated in 16S rRNA gene phylogeny. Two phylotypes were affiliated to the same SLOTU when the sequence similarity was above 99%. GLOTU and PLOTU corresponded to groups of SLOTU defined on the basis of the current taxonomy (http://www.ncbi.nlm.nih.gov/taxonomy).

### Diversity and dynamics data analysis

As bacteriological data were collected at least once a week but at various days of age in the recruited infants, we reported them as a function of age using a week-based scale. For each infant, the mean number of OTUs was computed for every week of age starting from delivery. The maximal number of species observed over the stool samples was also computed for each infant.

Semi-quantitative scoring was performed by visual inspection for each OTU using a four-point scale: 0 - no bacteria, 1 - few bacteria (+/weak band intensity), 2 - rather numerous bacteria (++/moderate band intensity), 3 numerous bacteria (+++/bright band intensity) as previously described by Roudière et al. (2009) (24). For each neonate, a semi-quantitative score was calculated per week for each GLOTU corresponding to mean semi-quantitative scores of the OTUs affiliated to the GLOTU. Finally, a mean semi-quantitative score was assessed per week for each GLOTU in the colonized population. The maximal semi-quantitative score observed for each GLOTU in each infant per week was also computed and a mean maximal score was calculated for the colonized patients. Colonization by an OTU was defined as either transient or persistent over the study period based on the number of positive samples (≤50% and >50%, respectively) for the considered OTU after the initial OTU recovery.

### Nucleotide sequences accession number

The partial sequences of 16S rRNA gene obtained in this study have been deposited in the GenBank database under accession numbers (*They will be provided upon acceptance of the article*).

## Additional files

Additional file 1: Table S1. Qualitative and semi-quantitative characteristics of gut colonization in the 30 preterm infants included in the study. Additional file 2:
**Nucleotide sequences of the TTGE bands (fasta format).**
